# What is the physical origin of the gradient flow structure of variational fracture models?

**DOI:** 10.1098/rsta.2023.0297

**Published:** 2024-07-15

**Authors:** Masato Kimura, Takeshi Takaishi, Yoshimi Tanaka

**Affiliations:** ^1^ Faculty of Mathematics and Physics, Kanazawa University, Kanazawa, Japan; ^2^ Musashino University, Tokyo, Japan; ^3^ Kanazawa Gakuin University, Kanazawa, Japan

**Keywords:** fracture mechanics, phase field model, variational fracture model

## Abstract

We investigate a physical characterization of the gradient flow structure of variational fracture models for brittle materials: a Griffith-type fracture model and an irreversible fracture phase field model. We derive the Griffith-type fracture model by assuming that the fracture energy in Griffith’s theory is an increasing function of the crack tip velocity. Such a velocity dependence of the fracture energy is typically observed in polymers. We also prove an energy dissipation identity of the Griffith-type fracture model, in other words, its gradient flow structure. On the other hand, the irreversible fracture phase field model is derived as a unidirectional gradient flow of a regularized total energy. We have considered the time relaxation parameter a mathematical approximation parameter, which we should choose as small as possible. In this research, however, we reveal the physical origin of the gradient flow structure of the fracture phase field model (F-PFM) and show that the small time relaxation parameter is characterized as the rate of velocity dependence of the fracture energy. It is verified by comparing the energy dissipation properties of those two models and by analysing a travelling wave solution of the irreversible F-PFM.

This article is part of the theme issue ‘Non-smooth variational problems with applications in mechanics’.

## 1. Introduction

This article considers variational fracture models for quasi-static crack propagation in a brittle material, especially a variant of the Griffith-type fracture model and an irreversible fracture phase field model (F-PFM). We also discuss their energy dissipation properties and the physical characterization of a small time relaxation parameter in the variational fracture model, that is, α>0 in (1.1)

Bourdin *et al*. [1] and Karma *et al*. [2] initiated the phase field approach to model fracture phenomena. Then, it is widely used for numerical studies of the dynamics of fracture under complex geometries and conditions in two-dimensional or three-dimensional, such as fractures in thermoelasticity [3–6], viscoelasticity [5,7], crack nucleation [8,9] and cracking phenomena with other physical and chemical effects [5,10,11].

**Figure 1.**
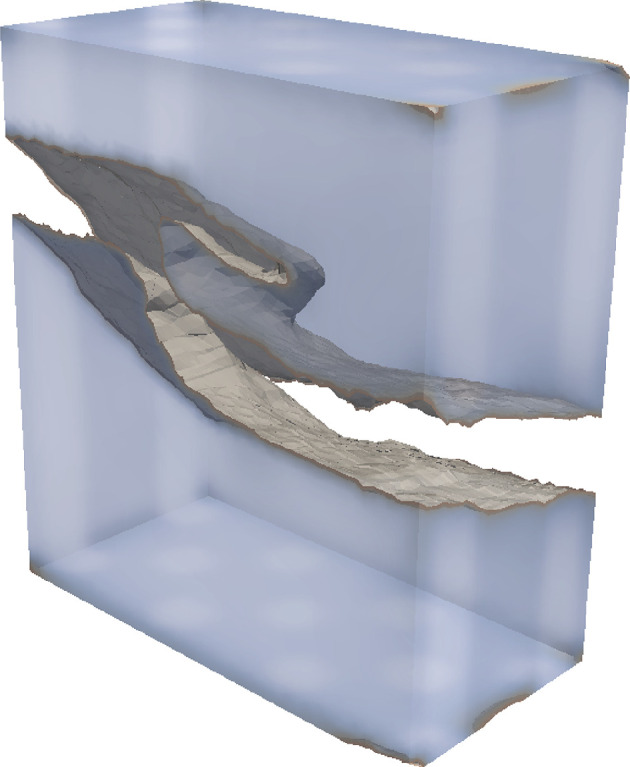
An example of fracture simulations by three-dimensional F-PFM.

The phase field model is a diffused interface approach to the crack problem, i.e. instead of describing the crack as a sharp boundary, a smooth phase field variable (damage field variable) is introduced over the material region. The following phase field model for fracture phenomena (which is denoted by F-PFM in this paper) was proposed in [5,12]:


(1.1) $$\left\{ \begin{array}{l} -\text{div}\left({\left(1-z\right)}^{2}\sigma \left[u\right]\right)=f\left(t\right),\\ \alpha \frac{\partial z}{\partial t}={\left(\varepsilon \;\text{div}\left(G_{c}\nabla z\right)-\frac{G_{c}}{\varepsilon }z+\left(1-z\right)\sigma \left[u\right]:e\left[u\right]\right)}_{+}, \end{array}\right.$$

where $u(x,t)\in {\mathbb{R}}^{d}$ ( d=2,3 ) denotes a displacement and z(x,t)∈[0,1] denotes a phase field variable for the crack position as z≈1 for the cracked region and z≈0 for the undamaged region. The phase field z is often called a damage variable. We denote strain and stress tensors by e[u] and σ[u] (see detail in §2a) and the fracture energy (the critical energy release rate) of the material by $G_{c}>0$ . The parameters α and ε are small positive real numbers related to regularizations in time and space, respectively. As a crack cannot be healed itself, we take the positive part ${\left(\;\right)}_{+}$ of the right-hand side of the second equation, where ${\left(a\right)}_{+}=\max(a,0)$ . The use of the positive part function guarantees the irreversibility of the crack propagation: $\frac{\partial z}{\partial t}\geq 0$ . Figure 1 shows an example of a finite element simulation of a complex fracture geometry by the three-dimensional F-PFM. See more detail in §4a and also [5].

**Figure 2.**
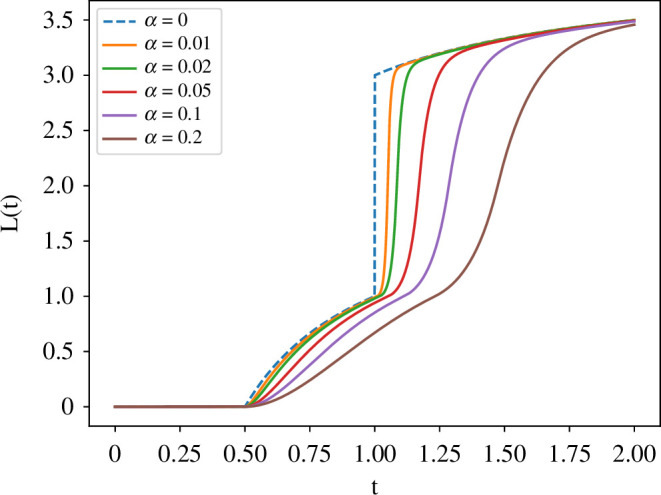
Numerical solutions of (3.7) for α=0.01−0.2 with G(l,t)=t(2−||l−1|−1|) and $G_{c}=1$ . The broken line shows the limit profile of L(t) as α→0 , which has a sudden jump at t=1 .

As shown in [5–7,12], the F-PFM successfully modelled various fracture phenomena with energy consistency. The F-PFM includes two artificial small positive parameters ε and α , which relate to the space regularization and the time relaxation, respectively. Roughly speaking, the crack tip singularity of the stress field is regularized by ε , and the ‘sudden jump’ singularity (see figure 2) of the crack propagation is regularized by α . These regularizations enable us to get a stable numerical crack propagation. However, the physical characterization of these small parameters has yet to be well studied.

This paper aims to clarify the physical characterization of the parameter α>0 in the F-PFM. As shown in Kimura *et al*. [5], the F-PFM satisfies an energy dissipation identity (4.3) and α becomes a coefficient of the dissipation term.

On the other hand, forming the process zone near the crack tip/edge causes such energy dissipation, and it is experimentally observed as a velocity dependence of the fracture energy (see §3a for details). To clarify the connection between the energy dissipation and the velocity dependence of the fracture energy, we consider a Griffith-type fracture model. Then, we reveal that the velocity-dependent fracture energy causes energy dissipation. The obtained energy dissipation identity represents its gradient flow structure, which resembles that of the F-PFM. Through such mathematical evidence, we systematically explain that the parameter α in the F-PFM has a clear physical meaning as the rate of the velocity dependence of the fracture energy.

The outline of this paper is as follows. §2 briefly reviews the energy dissipation identities in the classical Griffith theory and the variational fracture theory when the crack path is prescribed. Then, in §3, we consider Griffith’s crack propagation model with the velocity-dependent fracture energy and prove that it can be described as a well-posed initial value problem of an ODE, and satisfies a natural energy dissipation identity. In §4a, we will see that the gradient flow structure of the F-PFM implies an energy dissipation identity that resembles one of the ODE models in §3. Furthermore, we investigate the regularized fracture energy of the F-PFM by considering a travelling wave solution in §4b. Finally, in the last section we will give concluding remarks and open questions.

## 2. Quasi-static variational fracture theory

### (a) Crack problem in linear elasticity

We first consider a crack problem in static linear elasticity. We omit details of notation and mathematical assumptions here and refer to §2 of [5] for more precise definitions and mathematical settings. In this article, for simplicity, we often abbreviate the space variable x , e.g. u(t) means u(t)=u(x,t) or u(t)=u(⋅,t) . Let Ω be a bounded Lipschitz domain in ${\mathbb{R}}^{d}\;(d=2,3)$ , which represents an elastic body. We suppose that its boundary Γ:=∂Ω is decomposed into two portions $\Gamma_{D}$ and $\Gamma_{N}=\Gamma \setminus \Gamma_{D}$ , where $\Gamma_{D}$ is a nonempty (i.e. the (d−1) -dimensional volume of $\Gamma_{D}$ is positive) Lipschitz portion of Γ with the Dirichlet boundary condition, and a surface traction boundary condition is assumed on $\Gamma_{N}$ .

For each time t∈[0,T] , we consider a body force $f\left(t\right)\in L^{2}\left(\Omega ;R^{d}\right)$ and a surface traction $q\left(t\right)\in L^{2}\left(\Gamma_{N};R^{d}\right)$ . We also suppose that a given boundary displacement g(t) on $\Gamma_{D}$ can be extended on $\bar{\Omega }$ as $g\left(t\right)\in H^{1}\left(\Omega ;R^{d}\right)$ .

We suppose a crack $\Sigma \in C_{0}$ in Ω , where $C_{0}$ is an admissible set of cracks (see appendix B and also §2 of Alifian *et al*. [13]). We denote the length/area (for d=2 / d=3 ) of the crack Σ by |Σ| . We consider the following boundary value problem of linear elasticity in the cracked domain Ω∖Σ :


(2.1) $$\left\{ \begin{array}{llll} -\text{div}\;\sigma \left[u\right]=f\left(t\right)\;\;\;\; & \text{in}\;\Omega \setminus \Sigma , & & \\ \sigma \left[u\right]\nu =q\left(t\right) & \text{on}\;\Gamma_{\text{N}},\\ \sigma \left[u\right]\nu =0 & \text{on}\;\Sigma^{\pm },\\ u=g\left(t\right) & \text{on}\;\Gamma_{\text{D}}, \end{array}\right.$$

where the unknown function $u\left(x\right)={\left(u_{1}\left(x\right),\ldots ,u_{d}\left(x\right)\right)}^{\text{T}}\in R^{d}$ denotes a small displacement on Ω∖Σ . The strain tensor is defined by $e[u](x)=(e_{ij}[u](x))\in {\mathbb{R}}_{\text{sym}}^{d\times d}$ , $e_{ij}[u]:=\frac{1}{2}\left(\frac{\partial u_{i}}{\partial x_{j}}+\frac{\partial u_{j}}{\partial x_{i}}\right)$ , where ${\mathbb{R}}_{\text{sym}}^{d\times d}$ denotes the space of real-valued symmetric d×d matrices. We use the Einstein summation convention for spatial indices. The elasticity tensor $C=\left(c_{ijkl}\right)$ is assumed to satisfy the symmetry condition $c_{ijkl}=c_{klij}=c_{jikl}$ , and the positivity condition: $c_{ijkl}\;\xi_{ij}\;\xi_{kl}\;\geq \;c_{*}\;|\xi {|}^{2}\;\left(\xi \in R_{\text{sym}}^{d\times d}\right)$ , where $|\xi |:=\sqrt{\xi_{ij}\;\xi_{ij}}$ and $c_{*}>0$ . The stress tensor is denoted by $\sigma [u](x)=(\sigma_{ij}[u](x))\in {\mathbb{R}}_{\text{sym}}^{d\times d}$ and is defined as σ[u]:=Ce[u] i.e. $\sigma_{ij}\left[u\right]\left(x\right):=c_{ijkl}e_{kl}\left[u\right]\left(x\right)$ .

Under the above assumptions, for each t∈[0,T] , there exists a weak solution to (2.1), and we denote it by $u\left(t;\Sigma \right):\Omega \setminus \Sigma \to R^{d}$ . It is known that the following variational principle gives u(t;Σ) :


(2.2) $$u\left(t;\Sigma \right)=\underset{v\in V\left(g\left(t\right);\Sigma \right)}{\text{argmin}}E_{el}\left(t,v;\Sigma \right),$$

where $V\left(g;\Sigma \right):=\left\{v\in H^{1}\left(\Omega \setminus \Sigma ;R^{d}\right);\left(v-g\right){|}_{\Gamma_{\text{D}}}=0\right\}$ for $g\in H^{1}\left(\Omega ;R^{d}\right)$ , and


(2.3) $$E_{el}\left(t,v;\Sigma \right):=\frac{1}{2}\int_{\;\;\Omega \setminus \Sigma }\sigma \left[v\right]:e\left[v\right]\;dx-\int_{\;\;\Omega }f\left(t\right)\cdot v\;dx-\int_{\;\;\Gamma_{\text{N}}}q\left(t\right)\cdot v\;ds$$

represents the elastic energy of a displacement $v\in H^{1}\left(\Omega \setminus \Sigma ;R^{d}\right)$ including the body and surface forces at time t . Then, the elastic energy in the cracked body Ω∖Σ at time t is given as


(2.4) $$E_{el}^{*}\left(t;\Sigma \right):=\underset{v\in V\left(g\left(t\right);\Sigma \right)}{min}E_{el}\left(t,v;\Sigma \right)=E_{el}\left(t,u\left(t;\Sigma \right);\Sigma \right),$$

and it is known (e.g. [14]) that


(2.5) $$E_{el}^{*}\left(t;\Sigma \right)\geq E_{el}^{*}\left(t;\overset{˜}{\Sigma }\right)\;\;\;\;\text{holds, if}\;\;\;\;\Sigma \subset \overset{˜}{\Sigma }\in C_{0}.$$

When we fix the crack Σ and the given loads (g(t),f(t),q(t)) change in time sufficiently smoothly, the following energy conservation property holds


(2.6) $$\frac{d}{dt}E_{el}^{*}\left(t;\Sigma \right)=\overset{˙}{F}\left(t,u\left(t;\Sigma \right);\Sigma \right),$$

where


(2.7) $$\overset{˙}{F}\left(t,v;\Sigma \right):=\int_{\;\;\Gamma_{\text{D}}}\frac{\partial g}{\partial t}\left(t\right)\cdot \left(\sigma \left[v\right]\nu \right)\;ds-\int_{\;\;\Omega \setminus \Sigma }\frac{\partial f}{\partial t}\left(t\right)\cdot v\;dx-\int_{\;\;\Gamma_{\text{N}}}\frac{\partial q}{\partial t}\left(t\right)\cdot v\;ds.$$

The three terms on the right-hand side of (2.7) represent the rates of energy injection for a displacement v through the boundary displacement g(t) , the body force f(t) and the surface traction q(t) , respectively. Using the integration by parts formula under suitable regularity assumptions, we can derive the energy identity (2.6) (see also §2.3 of Kimura *et al.* [5]).

### (b) Energy profile and energy release rate along a given crack path

In the pioneering work by Griffith [15], he constructed an energetic fracture theory under the assumption that a crack path is given and that crack evolution is continuous in time. Refer to appendix B for the precise definitions of a crack path ${\left\{\Sigma_{p}\left(l\right)\right\}}_{l_{0}\leq l\leq l_{1}}$ and a crack evolution ${\left\{\Sigma \left(t\right)\right\}}_{t_{0}\leq t\leq t_{1}}$ .

For a given crack path ${\left\{\Sigma_{p}\left(l\right)\right\}}_{l_{0}\leq l\leq l_{1}}\subset C_{0}$ , which is parametrized by $l=|\Sigma_{p}\left(l\right)|$ , we define $E\left(l,t\right):=E_{el}^{*}\left(t;\Sigma_{p}\left(l\right)\right)$ for $l\in \left[l_{0},l_{1}\right]$ , and refer to the function l↦E(l,t) as an energy profile. If the energy profile E(l,t) is of $C^{1}$ -class in l , then $G\left(l,t\right):=-\frac{\partial E}{\partial l}\left(l,t\right)$ is called an energy release rate per unit length/area of the crack evolution. From (2.5), it follows that E(l,t) is non-increasing in l and G(l,t)≥0 holds. From (2.6), we also have


(2.8) $$\frac{\partial E}{\partial t}\left(l,t\right)=\overset{˙}{F}\left(t,u\left(t;\Sigma_{p}\left(l\right)\right);\Sigma_{p}\left(l\right)\right).$$

### (c) Griffith theory

According to [16–18], the classical Griffith theory is summarized as follows. We suppose that ${\left\{\Sigma \left(t\right)\right\}}_{t_{0}\leq t\leq t_{1}}$ is a smooth, continuous crack evolution in Ω under a given boundary condition g(t) , and ${\left\{\Sigma_{p}\left(l\right)\right\}}_{l_{0}\leq l\leq l_{1}}$ is the corresponding crack path (proposition B.3). Then, there exists $G_{c}>0$ such that L(t):=|Σ(t)| satisfies the following conditions:


(2.9) $$\left\{ \begin{array}{l} L^{\prime }\left(t\right)\geq 0\;\;\;\;\text{(Irreversibility)}\\ G\left(L\left(t\right),t\right)\leq G_{c}\;\;\;\;\left({\text{Griffit}}^{\prime }\text{s}\;\text{Criterion}\right)\\ L^{\prime }\left(t\right)\left(G_{c}-G\left(L\left(t\right),t\right)\right)=0\;\;\;\;\text{(Energy Conservation)} \end{array}\right.$$

for $t\in [t_{0},t_{1}]$ , where $G_{c}$ is a material property called a fracture energy (or a critical energy release rate). The third condition of (2.9) represents the conservation of a total energy


(2.10) $$E_{tot}^{*}\left(t;\Sigma \right):=E_{el}^{*}\left(t;\Sigma \right)+G_{c}|\Sigma |.$$

From (2.8) and $G\left(l,t\right):=-\frac{\partial E}{\partial l}\left(l,t\right)$ , we have


$$\frac{d}{dt}E_{tot}^{*}(t;\Sigma (t))=\frac{d}{dt}(E(L(t),t)+G_{c}L(t))=L^{\prime }(t)(G_{c}-G(L(t),t))+\dot{F}(t,u(t;\Sigma (t));\Sigma (t)).$$

This implies the following energy conservation law:


$$\frac{d}{dt}E_{tot}^{*}\left(t;\Sigma \left(t\right)\right)=\overset{˙}{F}\left(t,u\left(t;\Sigma \left(t\right)\right);\Sigma \left(t\right)\right),$$

provided the third condition of (2.9) holds.

## 3. Crack propagation model with velocity-dependent fracture energy

### (a) Velocity-dependent fracture energy

Many experiments [19–22] on metals, ceramics and polymers have revealed that the measured fracture energy (or, equivalently, critical stress intensity factor) depends on crack velocity. We denote the crack velocity-dependent fracture energy by $G_{c}^{*}\left(V\right)$ , where V≥0 is the two-dimensional crack tip velocity and the normal component (i.e. normal to the crack edge) of the three-dimensional crack edge velocity.

The physical origin of the V -dependence is the formation of the so-called process zone around the crack tip [19]. A process zone has an intermediate spatial scale (far larger than the atomic scale and far smaller than the specimen size), and some dissipative processes occur there. The size of the process zone and the intensity of the energy dissipation change with V , and we can macroscopically measure those dependencies on V as a V -dependence of the fracture energy.

**Figure 3.**
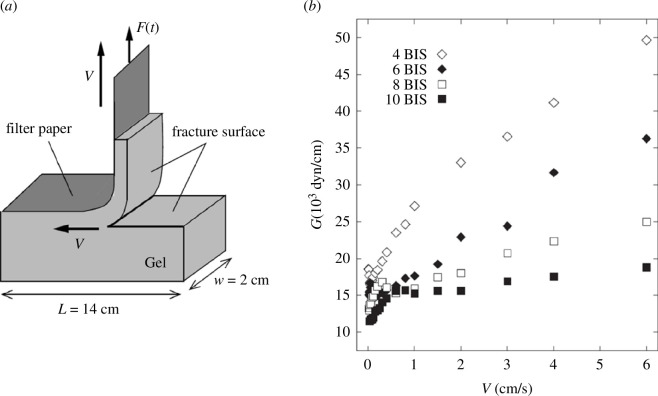
Crack velocity ( V ) dependence of fracture energy ( G ) of chemically cross-linked acrylamide hydrogels is shown in (b) measured by a sort of tearing test (*a*), taken from Tanaka *et al*. [23] with kind permission of *The European Physical Journal*: The difference in plot symbols represents the difference in cross-link density. As the cross-link density increases, G(V) gets lower. The data of G(V) show slightly upper convex behaviour but almost linear for larger V .

Usually, $G_{c}^{*}\left(V\right)$ increases with V (the faster deformations cause the larger dissipations). Especially, gel materials, cross-linked polymer networks swollen with solvent, tend to show a simple, almost linearly increasing behaviour, as seen in figure 3 [23] (also [24]). In the following lines, we assume $G_{c}^{*}\left(V\right)$ is a strictly increasing function of V . However, it is experimentally possible that $G_{c}^{*}\left(V\right)$ shows a negative slope or a drastic drop in a particular V region if the fracture mechanism qualitatively changes in the V region (e.g. brittle–ductile transition [19]).

### (b) An ODE model and energy dissipation

**Figure 4.**
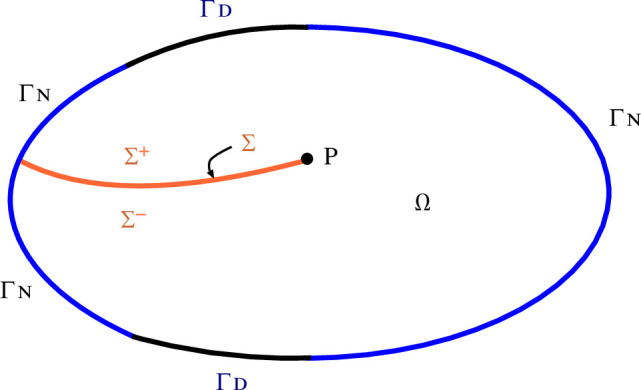
A cracked domain Ω∖Σ with a Dirichlet boundary $\Gamma_{\text{D}}$ and a Neumann boundary $\Gamma_{\text{N}}$ .

In this section, we set d=2 and suppose the crack has a single tip P , as in figure 4. Then, $V=L^{\prime }(t)$ denotes the crack propagation velocity. We assume the following condition on $G_{c}^{*}\left(V\right)$


(3.1) $$\left\{ \begin{array}{l} G_{c}^{*}\left(V\right)=G_{c}+\alpha^{*}\left(V\right)\;\;\;\;\left(V\in \left[0,\infty \right)\right),\;\;\;\;G_{c}>0,\\ \alpha^{*}\text{is a strictly increasing continuous function on}\;\left[0,\infty \right)\;\text{with}\;\alpha^{*}\left(0\right)=0. \end{array}\right.$$

When the fracture energy depends on the crack tip velocity as $G_{c}^{*}\left(V\right)$ , the Griffith model 2.9 becomes


(3.2) $$\left\{ \begin{array}{l} V\geq 0,\\ G\leq G_{c}^{*}\left(V\right),\\ V\left(G_{c}^{*}\left(V\right)-G\right)=0, \end{array}\right.$$

where G=G(L(t),t) . We call (3.2) a Griffith-type fracture model with velocity-dependent fracture energy. We have the following theorem.

***Theorem 3.1.*** *Under the condition* (3.1), *the velocity-dependent fracture energy model* (3.2) *is equivalent to*


(3.3) $$\alpha^{*}\left(V\right)={\left(G-G_{c}\right)}_{+}.$$

*It is also equivalent to*


(3.4) $$V=\beta^{*}\left(G-G_{c}\right),$$

*where* $\beta^{*}\left(s\right):={\left(\alpha^{*}\right)}^{-1}\left(s\right)$ *for* s≥0 *and* $\beta^{*}(s):=0$ *for* s<0 .

*Proof*. Under the condition (3.1), $\alpha^{*}(V)\geq 0$ holds if and only if V≥0 holds, and $\alpha^{*}(V)=0$ holds if and only if V=0 holds. So, (3.2) is equivalent to


(3.5) $$\left\{ \begin{array}{l} \alpha^{*}\left(V\right)\geq 0,\\ G_{c}^{*}\left(V\right)-G\geq 0,\\ \alpha^{*}\left(V\right)\left(G_{c}^{*}\left(V\right)-G\right)=0. \end{array}\right.$$

Then, applying (A.1) of lemma A.1, we obtain that (3.5) is equivalent to


$$\alpha^{*}\left(V\right)={\left(\alpha^{*}\left(V\right)-\left(G_{c}^{*}\left(V\right)-G\right)\right)}_{+}={\left(G-G_{c}\right)}_{+}.$$

The equivalency to the alternative form (3.4) is quickly confirmed. ∎

**Remark 3.2.** From theorem 3.1, the Griffith-type model (3.2) with initial crack length $l_{0}$ is equivalent to the following initial value problem of an ODE


(3.6) $$\left\{ \begin{array}{l} L^{\prime }\left(t\right)=\beta^{*}\left(G\left(L\left(t\right),t\right)-G_{c}\right)\;\;\;\;\left(t\geq t_{0}\right),\\ L\left(t_{0}\right)=l_{0}. \end{array}\right.$$

The function G(l,t) is assumed to be continuous in (l,t) and locally Lipschitz in l . If $\beta^{*}$ is also locally Lipschitz (e.g. this is true if $\alpha^{*}\in C^{1}([0,\infty ))$ and ${\left(\alpha^{*}\right)}^{\prime }\left(V\right)>0$ for V≥0 ), then from the Cauchy–Lipschitz theorem, it follows that there exists a unique solution to (3.6) locally in time.

**Remark 3.3.** When $\alpha^{*}(V)$ is a linear function as $\alpha^{*}(V)=\alpha V$ with α>0 , then $\beta^{*}(s)=\frac{1}{\alpha }{\left(s\right)}_{+}$ holds. In this case, (3.6) becomes


(3.7) $$\left\{ \begin{array}{l} \alpha L^{\prime }\left(t\right)={\left(G\left(L\left(t\right),t\right)-G_{c}\right)}_{+}\;\;\;\;\left(t\geq t_{0}\right),\\ L\left(t_{0}\right)=l_{0}. \end{array}\right.$$

In figure 2, we draw numerical solutions of (3.7) for different α∈[0.01,0.2] with $G_{c}=1$ and an artificially given energy release rate function G(l,t):=t(2−||l−1|−1|) . The broken line in the figure shows the limit profile of L(t) as α→0 , which has a sudden jump at t=1 . In other words, the small parameter α>0 has the role of regularizing singularity in time. Such a sudden jump in the crack propagation is described in the framework of the variational fracture theory by Francfort and Marigo [14,17]. However, the limit profile in the figure captures a slightly different behaviour from the original variational fracture theory [14]. It corresponds to a localized Franctort–Marigo model [13,25]. We no longer discuss this issue in this paper, but it will be discussed in our forthcoming paper intensively.

***Theorem 3.4.*** *(Energy dissipation identity). Under the settings in* §2*, we suppose that the energy profile* E(l,t) *satisfies* $E\in C^{1}\left(\left[l_{0},l_{1}\right]\times \left[t_{0},t_{1}\right]\right)$ *and* (2.8), *and that* $L\in C^{1}\left(\left[t_{0},t_{1}\right]\right)$ *is a solution of* (3.6) *on* $\left[t_{0},t_{1}\right]$ . *We define* $\Sigma \left(t\right):=\Sigma_{p}\left(L\left(t\right)\right)$ *and* $V\left(t\right):=L^{\prime }\left(t\right)$ . *Then, it satisfies the following energy dissipation identity:*


(3.8) $$\frac{d}{dt}E_{tot}^{*}\left(t;\Sigma \left(t\right)\right)=-\alpha^{*}\left(V\left(t\right)\right)V\left(t\right)+\overset{˙}{F}\left(t,u\left(t;\Sigma \left(t\right)\right);\Sigma \left(t\right)\right).$$

*In particular, when* $\alpha^{*}(V)=\alpha V$ ,


$$\frac{d}{dt}E_{tot}^{*}\left(t;\Sigma \left(t\right)\right)=-\alpha |V\left(t\right){|}^{2}+\overset{˙}{F}\left(t,u\left(t;\Sigma \left(t\right)\right);\Sigma \left(t\right)\right).$$

*Proof.* Since $E_{tot}^{*}\left(t;\Sigma \left(t\right)\right)=E\left(L\left(t\right),t\right)+G_{c}L\left(t\right)$ , we have


$$\begin{array}{ll} \frac{d}{dt}E_{tot}^{*}\left(t;\Sigma \left(t\right)\right) & =\frac{d}{dt}\left(E\left(L\left(t\right),t\right)+G_{c}L\left(t\right)\right)\\ & =\left(\frac{\partial E}{\partial l}\left(L\left(t\right),t\right)+G_{c}\right)L^{\prime }\left(t\right)+\frac{\partial E}{\partial t}\left(L\left(t\right),t\right)\\ & =-\left(G\left(L\left(t\right),t\right)-G_{c}\right)V\left(t\right)+\overset{˙}{F}\left(t,u\left(t;\Sigma \left(t\right)\right);\Sigma \left(t\right)\right). \end{array}$$

Hence, (3.8) follows from $\left(G(L(t),t)-G_{c})V(t)=\alpha^{*}(V(t))V(t\right)$ , which we derive from (3.3). ∎

### (c) Discussion

In this section, we studied the Griffith-type model (3.2) with a velocity-dependent fracture energy $G_{c}^{*}\left(V\right)=G_{c}+\alpha^{*}\left(V\right)$ , typically observed in polymer materials. Theorem 3.1 proved that the Griffith model with the velocity-dependent fracture energy (3.2) is equivalent to the ODE model (3.6). In particular, when the fracture energy linearly depends on the velocity $G_{c}^{*}\left(V\right)=G_{c}+\alpha V$ , then it is written in the form: $\alpha L^{\prime }\left(t\right)={\left(G\left(L\left(t\right),t\right)-G_{c}\right)}_{+}$ and satisfies the energy dissipation identity: $\frac{d}{dt}E_{tot}^{*}\left(t;\Sigma \left(t\right)\right)=-\alpha |V\left(t\right){|}^{2}+\overset{˙}{F}$ . As we will see in §4a, the above energy dissipation structure closely resembles that of the F-PFM.

## 4. Irreversible F-PFM

### (a) F-PFM and energy dissipation identity

**Figure 5.**
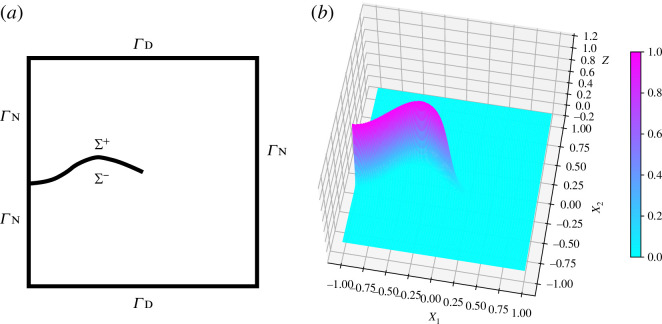
A crack Σ in a two-dimensional rectangular domain Ω and a corresponding phase field variable z(x) are illustrated in the (*a*) and (*b*) figures, respectively.

This section briefly introduces an irreversible F-PFM based on [5,12]. We consider a smooth phase field function z(x,t) to represent an approximate profile of the crack Σ(t) (figure 5). We assume that 0≤z(x,t)≤1 and z(x,t)≈1 around crack Σ(t) , and that z(x,t)≈0 for the other region. The phase field z is also called a damage variable, representing a relative amount of the accumulated damage in the elastic material. With the damage variable z , $\tilde{C}:={\left(1-z\right)}^{2}C$ gives the damaged elasticity tensor, where C denotes the original non-damaged elasticity tensor.

The F-PFM is described as the following initial and boundary value problem of an elliptic–parabolic system of partial differential equations


(4.1) $$\left\{ \begin{array}{ll} -\text{div}\left({\left(1-z\right)}^{2}\sigma \left[u\right]\right)=f\left(t\right)\;\;\;\; & \text{in}\;\Omega \times \left[0,T\right],\\ \alpha \frac{\partial z}{\partial t}={\left(\varepsilon \;\text{div}\left(G_{c}\nabla z\right)-\frac{G_{c}}{\varepsilon }z+\left(1-z\right)\sigma \left[u\right]:e\left[u\right]\right)}_{+}\;\;\;\; & \text{in}\;\Omega \times \left(0,T\right],\\ u=g\left(t\right)\;\;\;\; & \text{on}\;\Gamma_{\text{D}}\times \left[0,T\right],\\ \sigma \left[u\right]\nu =q\left(t\right)\;\;\;\; & \text{on}\;\Gamma_{\text{N}}\times \left[0,T\right],\\ \frac{\partial z}{\partial \nu }=0\;\;\;\; & \text{on}\;\Gamma \setminus \Gamma_{\text{N}}^{1}\times \left[0,T\right],\\ z=0\;\;\;\; & \text{on}\;\Gamma_{\text{N}}^{1}\times \left[0,T\right],\\ z{|}_{t=0}=z^{0}\;\;\;\; & \text{in}\;\Omega . \end{array}\right.$$

We suppose that q(x,t)=0 for $x\in \Gamma_{\text{N}}^{0}\subset \Gamma_{N}$ and t∈[0,T] , and set $\Gamma_{\text{N}}^{1}:=\Gamma_{\text{N}}\setminus \Gamma_{\text{N}}^{0}$ . The second equation, a nonlinear parabolic equation of z , describes the crack propagation. The parameters α and ε are small positive real numbers related to regularizations in time and space, respectively. The positive part of the second equation’s right-hand side guarantees the crack propagation’s irreversibility.

Instead of the elastic energy $E_{el}(t,v;\Sigma )$ of (2.3) and the surface energy $G_{c}|\Sigma |$ , we define the following regularized elastic energy $\varepsilon_{el}\left(t,v,z\right)$ and surface energy $\varepsilon_{s}\left(z\right)$ applying the Ambrosio–Tortorelli approximation [26]


$$\begin{array}{c} \varepsilon_{el}\left(t,u,z\right):=\frac{1}{2}\int_{\;\;\Omega }{\left(1-z\right)}^{2}\sigma \left[u\right]:e\left[u\right]\;dx-\int_{\;\;\Omega }f\left(t\right)\cdot u\;dx-\int_{\;\;\Gamma_{\text{N}}}q\left(t\right)\cdot u\;ds,\\ \varepsilon_{s}\left(z\right):=\frac{1}{2}\int_{\;\;\Omega }G_{c}\left(\varepsilon |\nabla z{|}^{2}+\frac{1}{\varepsilon }z^{2}\right)\;dx. \end{array}$$

We set $V\left(g\right):=\left\{v\in H^{1}\left(\Omega ;R^{d}\right);\left(v-g\right){|}_{\Gamma_{\text{D}}}=0\right\}$ for $g\in H^{1}\left(\Omega ;R^{d}\right)$ . Similarly to the case of $E_{el}$ , we define


$$\begin{array}{c} u\left(t,z\right):=\underset{v\in V\left(g\left(t\right)\right)}{\text{argmin}}\;\varepsilon_{el}\left(t,v,z\right),\\ \varepsilon_{el}^{*}\left(t,z\right):=\underset{v\in V\left(g\left(t\right)\right)}{min}\varepsilon_{el}\left(t,v,z\right)=\varepsilon_{el}\left(t,u\left(t,z\right),z\right),\\ \varepsilon_{tot}^{*}\left(t,z\right):=\varepsilon_{el}^{*}\left(t,z\right)+\varepsilon_{s}\left(z\right). \end{array}$$

The F-PFM (4.1) is derived as a so-called irreversible gradient flow [27] of $E_{tot}^{*}\left(t,z\right)$ with respect to z


(4.2) $$\alpha \frac{\partial z}{\partial t}={\left(-\frac{\delta \varepsilon_{tot}^{*}}{\delta z}\right)}_{+}.$$

In [5], the following energy dissipation equality was shown. If (u(t),z(t)) is a sufficiently smooth solution of (4.1), then it satisfies


(4.3) $$\frac{d}{dt}\varepsilon_{tot}^{*}\left(t,z\left(t\right)\right)=-\alpha \int_{\;\;\Omega }{\left|\frac{\partial z}{\partial t}\right|}^{2}\;dx+\dot{F}\left(t,u\left(t\right),z\left(t\right)\right),$$

where


$$\dot{F}\left(t,u,z\right):=\int_{\;\;\Gamma_{\text{D}}}\frac{\partial g}{\partial t}\left(t\right)\cdot \left({\left(1-z\right)}^{2}\sigma \left[u\right]\nu \right)\;ds-\int_{\;\;\Omega }\frac{\partial f}{\partial t}\left(t\right)\cdot u\;dx-\int_{\;\;\Gamma_{\text{N}}}\frac{\partial q}{\partial t}\left(t\right)\cdot u\;ds.$$

As shown above, the F-PFM is derived based on the variational fracture theory [14,15], the Ambrosio–Tortorelli regularization [26], the unidirectional gradient flow [27] and consequently it exhibits a natural energy dissipation property (4.3). In contrast to the other crack propagation models, the F-PFM implicitly includes the crack path search and enables us to treat complex crack patterns even in three dimensions (figure 1).

On the other hand, from the viewpoint of physics, there are two open questions about the modelling of F-PFM. One is the physical characterization of the damage variable z and the spatial regularization parameter ε . Here, z and ε are introduced in the mathematical regularization technique [26], and their phyical substances have not been clarified yet.

The other open question is a physical characterization of the time relaxation parameter introduced in the gradient flow. This paper aims to clarify the physical meaning of the parameter α in the F-PFM. As we have discussed in §3, α in the ODE model (3.7) is characterized by the rate of velocity dependence of the fracture energy $G_{c}^{*}(V)$ : $\alpha =\frac{dG_{c}^{*}}{dV}$ . From the strong analogy between (3.7) and F-PFM, we expect to characterize the parameter α in F-PFM similarly. To strengthen this claim, we study the regularized fracture energy of the F-PFM by considering a travelling wave solution in the following §4b.

### (b) Travelling wave solution and velocity dependence of the fracture energy

**Figure 6.**
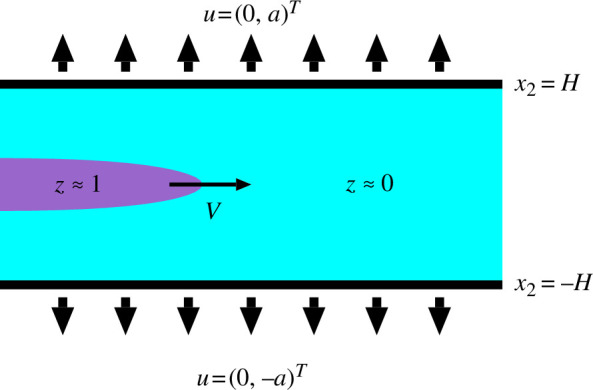
A travelling wave solution of the F-PFM in two dimensions.


In this section, we consider an infinite strip domain $\Omega_{H}:=R\times \left(-H,H\right)\subset R^{2}$ as shown in figure 6, and consider a travelling wave solution of the F-PFM in $\Omega_{H}$ . We set $\Gamma_{H}^{\pm }:=\{x={\left(x_{1},x_{2}\right)}^{T}\in {\mathbb{R}}^{2};\;x_{2}=\pm H\}$ . We consider the F-PFM in the strip domain $\Omega_{H}$ for t∈ℝ .

(4.4) $$\left\{ \begin{array}{ll} -\text{div}\left({\left(1-z\right)}^{2}\sigma \left[u\right]\right)=0\;\;\;\; & \text{in}\;\Omega_{H}\times R,\\ \alpha \frac{\partial z}{\partial t}={\left(G_{c}\left(\varepsilon \Delta z-\frac{z}{\varepsilon }\right)+\left(1-z\right)\sigma \left[u\right]:e\left[u\right]\right)}_{+}\;\;\;\; & \text{in}\;\Omega_{H}\times R,\\ u=\left(\begin{array}{c} 0\\ \pm a \end{array}\right)\;\;\;\; & \text{on}\;\Gamma_{H}^{\pm }\times R,\\ \partial_{2}z=0\;\;\;\; & \text{on}\;\Gamma_{H}^{\pm }\times R, \end{array}\right.$$

α>0. where a>0 .

This geometry corresponds to the so-called ‘pure-shear geometry’ experimentally realized. Moreover, the fracture energy’s velocity dependence for rubbers and gels is measured in that experimental setting [28]. The experimental system is a long rectangular plate (−W,W)×(−H,H)×(0,b) , where b>0 is the thickness of this plate. We realize a plane stress state in condition b<<H<<W . An initial crack is made from the centre of the left edge along the horizontal axis ( $x_{1}$ axis). We suppose that a pair of constant vertical boundary displacements of ±a at the upper and bottom edges of the system, i.e. $u_{2}(x_{1},\pm H,t)=\pm a$ , is applied. The above boundary value problem (4.4) corresponds to the case W=∞ .

We suppose that there exists a travelling wave solution with a velocity V>0 in the direction of positive $x_{1}$ , i.e. there exists $\bar{u}:\bar{\Omega_{H}}\to R^{2}$ and $\bar{z}:\bar{\Omega_{H}}\to \left(0,1\right)$ and V>0 such that


$$u(x_{1},x_{2},t)=\bar{u}(x_{1}-Vt,x_{2})=\bar{u}(\xi ),\;\;\;\;z(x_{1},x_{2},t)=\bar{z}(x_{1}-Vt,x_{2})=\bar{z}(\xi ),$$

where we set a moving coordinate $\xi :={\left(x_{1}-Vt,x_{2}\right)}^{T}\in \bar{\Omega_{H}}$ . Then, since $\partial_{t}z(x,t)=-V\partial_{1}\bar{z}(\xi )$ , $\left(\bar{u},\bar{z},V\right)$ is a solution of the following system:


(4.5) $$\left\{ \begin{array}{ll} -\text{div}\left({\left(1-\bar{z}\right)}^{2}\sigma \left[\bar{u}\right]\right)=0\;\;\;\; & \text{in}\;\Omega_{H},\\ -\alpha V\partial_{1}\bar{z}=G_{c}\left(\varepsilon \Delta \bar{z}-\frac{\bar{z}}{\varepsilon }\right)+\left(1-\bar{z}\right)w\;\;\;\; & \text{in}\;\Omega_{H},\\ \bar{u}=\left(\begin{array}{c} 0\\ \pm a \end{array}\right)\;\;\;\; & \text{on}\;\Gamma_{H}^{\pm },\\ \partial_{2}\bar{z}=0\;\;\;\; & \text{on}\;\Gamma_{H}^{\pm }, \end{array}\right.$$

where we have defined the density of elastic energy w(ξ) by $w(\xi ):=\sigma [\bar{u}]:e[\bar{u}](\xi )$ for $\xi \in \bar{\Omega_{H}}$ . Additionally, we omitted the positive part of the second equation since the expected profile of the travelling wave solution is $z_{t}>0$ .

According to a number of our numerical experiments of the F-PFM, we expect a travelling wave solution that corresponds to the constant-velocity crack propagation, as shown in figure 6. Since equation (4.5) is shift-invariant in the direction of $\xi_{1}$ , for a solution $\left(\bar{u}(\xi ),\bar{z}(\xi ),V\right)$ , $\left(\bar{u}\left(\xi_{1}-c,\xi_{2}\right),\bar{z}\left(\xi_{1}-c,\xi_{2}\right),V\right)$ is also a solution of equation (4.5) for any c∈ℝ . We fix a solution $\left(\bar{u}(\xi ),\bar{z}(\xi ),V\right)$ in the following discussion.

For t>0 and a sufficiently small ε>0 , the increment of the regularized crack length during the time interval (0,t) is given by


$$L_{\varepsilon }\left(t\right):=\frac{1}{2}\int_{\;\;\Omega_{H}}\left\{\left(\varepsilon |\nabla z\left(x,t\right){|}^{2}+\frac{z{\left(x,t\right)}^{2}}{\varepsilon }\right)-\left(\varepsilon |\nabla z\left(x,0\right){|}^{2}+\frac{z{\left(x,0\right)}^{2}}{\varepsilon }\right)\right\}\;dx.$$

Since V should coincide with $L_{\varepsilon }^{\prime }\left(t\right)$ , it holds that $L_{\varepsilon }^{\prime }\left(t\right)\approx V$ . We also have


$$\begin{array}{ll} L_{\varepsilon }^{\prime }\left(t\right) & =\int_{\;\;\Omega_{H}}\left\{\varepsilon \nabla z\left(x,t\right)\cdot \nabla z_{t}\left(x,t\right)+\frac{1}{\varepsilon }z\left(x,t\right)z_{t}\left(x,t\right)\right\}\;dx\\ & =-\int_{\;\;\Omega_{H}}\left(\varepsilon \Delta z\left(x,t\right)-\frac{1}{\varepsilon }z\left(x,t\right)\right)z_{t}\left(x,t\right)\;dx\\ & =V\int_{\;\;\Omega_{H}}\left(\varepsilon \Delta \bar{z}-\frac{\bar{z}}{\varepsilon }\right)\partial_{1}\bar{z}\;d\xi . \end{array}$$

Similarly, the increment of the elastic energy during the time interval (0,t) is estimated by


$$E_{\varepsilon }\left(t\right):=\frac{1}{2}\int_{\;\;\Omega_{H}}\left\{\left({\left(1-z\left(x,t\right)\right)}^{2}\sigma \left[u\left(\cdot ,t\right)\right]:e\left[u\left(\cdot ,t\right)\right]\right)-\left({\left(1-z\left(x,0\right)\right)}^{2}\sigma \left[u\left(\cdot ,0\right)\right]:e\left[u\left(\cdot ,0\right)\right]\right)\right\}\;dx,$$

and we have


$$\begin{array}{ll} E_{\varepsilon }^{\prime }\left(t\right) & =-\int_{\;\;\Omega_{H}}\left(1-z\left(x,t\right)\right)\partial_{t}z\left(x,t\right)\sigma \left[u\left(\cdot ,t\right)\right]:e\left[u\left(\cdot ,t\right)\right]dx\\ & +\int_{\;\;\Omega_{H}}{\left(1-z\left(x,t\right)\right)}^{2}\sigma \left[u\left(\cdot ,t\right)\right]:e\left[\partial_{t}u\left(\cdot ,t\right)\right]dx\\ & =V\int_{\;\;\Omega_{H}}\left(1-\bar{z}\left(\xi \right)\right)\partial_{1}\bar{z}\left(\xi \right)w\left(\xi \right)d\xi -\int_{\;\;\Omega_{H}}\text{div}{\left(1-z\left(x,t\right)\right)}^{2}\sigma \left[u\left(\cdot ,t\right)\right]\cdot \partial_{t}u\left(\cdot ,t\right)dx\\ & =V\int_{\;\;\Omega_{H}}\left(1-\bar{z}\right)w\partial_{1}\bar{z}d\xi \\ & =-V\int_{\;\;\Omega_{H}}\left\{\alpha V\partial_{1}\bar{z}+G_{c}\left(\varepsilon △\bar{z}-\frac{\bar{z}}{\varepsilon }\right)\right\}\partial_{1}\bar{z}\left(\xi \right)d\xi \end{array}$$

The increment of the total energy during the time interval (0,t) is given by $E_{\varepsilon }(t)+G_{c}L_{\varepsilon }(t)$ and we have the energy dissipation identity:


(4.6) $$\frac{d}{dt}\left(E_{\varepsilon }\left(t\right)+G_{c}L_{\varepsilon }\left(t\right)\right)=-\alpha \beta V^{2},$$

where $\beta :=\int_{\;\;\Omega_{H}}|\partial_{1}\bar{z}{|}^{2}\;d\xi >0$ .

Since the fracture energy (the critical energy release rate) $G_{c}>0$ is defined by the ratio of the released elastic energy per unit length of the propagating crack, we consider an effective fracture energy $G_{c}^{\varepsilon }$ for the travelling wave solution $\bar{z}(\xi )$ of the F-PFM: $G_{c}^{\varepsilon }:=-E_{\varepsilon }^{\prime }\left(t\right)/V$ . From the energy dissipation identity (4.6) and the above approximation $L_{\varepsilon }^{\prime }\left(t\right)\approx V$ , we obtain


(4.7) $$G_{c}^{\varepsilon }=G_{c}\frac{L_{\varepsilon }^{\prime }\left(t\right)}{V}+\alpha \beta V\approx G_{c}+\alpha \beta V.$$

The obtained formula (4.7) suggests that the time relaxation parameter α in the F-PFM corresponds to the rate of velocity dependence of the regularized fracture energy $\alpha \approx \beta^{-1}\frac{dG_{c}^{\varepsilon }}{dV}$ .

## 5. Discussion and concluding remarks

In this article, we studied the Griffith-type ODE model with velocity-dependent fracture energy (§3) and analysis of the travelling wave solution of the irreversible F-PFM (§4). In this section, we will discuss the physical meaning of these analyses and their conclusions.

As we saw in §3a, in the fracture of hydrogels, owing to the effect of the process zone formed at the crack tip, the fracture energy $G_{c}^{*}$ , that is, the energy per unit area that generates the crack surface, depends on the crack tip velocity V and becomes $G_{c}^{*}=G_{c}+\alpha^{*}\left(V\right)$ . In particular, in materials where the region away from the crack tip behaves elastically, and rate-dependent dissipation occurs only in the process zone near the crack tip, the V -dependence of fracture energy becomes approximately linear, i.e. $G_{c}^{*}=G_{c}+\alpha V$ [23]. Also, experimentally, α determined by measurements at a constant crack tip velocity is considered valid even for the general case where V is time-space-dependent. This idea is justified because the amount of dissipation in the process zone determining the value of α depends locally on space and time.

On the other hand, in §3b, we analysed the classical Griffith model (2.9) assuming V -dependent fracture energy $G_{c}^{*}=G_{c}+\alpha^{*}\left(V\right)$ . As a result, we obtained an ODE model (3.3) and proved the well-posedness of the model (remark 3.2). We can also write it in the form


(5.1) $$\alpha^{*}\left(L^{\prime }\left(t\right)\right)={\left(-\frac{\delta E_{tot}^{**}}{\delta L}\right)}_{+},$$

where $E_{tot}^{**}\left(L,t\right):=E\left(L,t\right)+G_{c}L$ and $\frac{\delta E_{tot}^{**}}{\delta L}=G_{c}-G\left(L,t\right)$ . This (5.1) represents an irreversible gradient flow structure induced from V -dependent fracture energy. In addition, in the case of linear dependence $\alpha^{*}(V)=\alpha V$ , it exhibits the similarity with the irreversible gradient flow structure (4.2) of F-PFM. It strongly suggests that the time constant parameter α of F-PFM is characterized by $\alpha \approx \frac{dG_{c}^{*}}{dV}$ .

Based on the above results, in §4b, we analysed the travelling wave solution of F-PFM that evolves at a constant speed and shape. As a result, the effective fracture energy $G_{c}^{\varepsilon }$ is also expressed as $G_{c}^{\varepsilon }\approx G_{c}+\alpha \beta V$ using a specific constant β>0 . It is observed that there is a region where z changes significantly near the crack tip in the F-PFM. It corresponds to the process zone in the actual fracture. Even in general cases, if essentially the same elastic and damage fields as in the travelling wave solution are formed around the crack tip, the obtained relation $\alpha \approx \beta^{-1}\frac{dG_{c}^{\varepsilon }}{dV}$ would apply to broader situations.

According to experimental measurements of some polymers, e.g. [23,24], the V -dependence is not always linear but exhibits several nonlinearities. In this study, we have established that the F-PFM corresponds to the case of linear V -dependence: $G_{c}^{*}\left(V\right)=G_{c}+\alpha V$ . This analysis suggests a further generalization of the F-PFM with a nonlinear V -dependent $G_{c}^{*}\left(V\right)=G_{c}+\alpha^{*}\left(V\right)$


(5.2) $$\alpha^{*}\left(\frac{\partial z}{\partial t}\right)={\left(-\frac{\delta E_{tot}^{*}}{\delta z}\right)}_{+},$$

with (3.1) as an analogy of (5.1). The model (5.2) is expected to be a potential mathematical model for crack propagation in polymers, which often exhibit nonlinear V -dependence of the fracture energy.

In conclusion, we revealed that the physical origin of the gradient flow structure of the variational fracture models is the velocity dependence of the fracture energy, which originated from the localized energy dissipation by the formation of the process zone around the crack tip.

## Data Availability

Data for figure 3 is taken from Tanaka *et al*. [23] with the kind permission of The European Physical Journal. Data for figure 2 has been uploaded as supplementary material [29].

## A. Lemma for the positive part

Let us define the positive part of c∈ℝ by ${\left(c\right)}_{+}:=\max(c,0)$ . We repeatedly use the following simple lemma concerning the positive part in our arguments.

**Lemma A.1**. For a, b∈ℝ , it holds that

(A 1)
$$a={\left(a-b\right)}_{+}⟺\left\{ \begin{array}{l} a\geq 0\\ b\geq 0\\ ab=0, \end{array}\right.$$

Alternatively, for a,c∈R , it holds that

(A 2)
$$a={\left(c\right)}_{+}⟺\left\{ \begin{array}{l} a\geq 0\\ a\geq c\\ a\left(a-c\right)=0. \end{array}\right.$$

*Proof*. As the relation ( A 1) is obtained from (A 2) by replacing b=a−c , we prove (A 2). Suppose $a={\left(c\right)}_{+}$ . Then a≥0 and a≥c hold. If a−c>0 , it implies $c<a={\left(c\right)}_{+}$ and $a={\left(c\right)}_{+}=0$ . Hence, the three conditions on the right-hand side of (A 2) are derived.

Conversely, if we suppose the three conditions on the right-hand side of (A 2), one of the following two cases holds: (i) 0<a=c , (ii) 0=a≥c , in both cases, we can quickly check that the condition $a={\left(c\right)}_{+}$ holds.∎

## B. Crack evolution and crack path

According to §2 of [13] (but the definition of $C_{0}$ is modified), we define admissible sets of cracks:

$$C:=\{\Sigma \subset \Omega ;\;\Omega \;\setminus \;\Sigma \;is\;open,\;\bar{\Sigma }\;\cap \;{\bar{\Gamma }}_{D}=\emptyset ,H^{d-1}\;(\Sigma )<\infty \},\;\;C_{0}:=\{\Sigma \in C;\;(B.1)\;\;holds\},$$

where $H^{d-1}$ is the (d−1) -dimensional Hausdorff measure and we set $|\Sigma |:=H^{d-1}\left(\Sigma \right)$ . For the unique solvability of a weak solution of (2.1), we suppose the following Korn’s inequality:

(B 1)
$${\;}^{\exists }C_{K}=C_{K}\left(\Omega ,\Gamma_{D},\Sigma \right)>0\;\text{s}\text{.t}\text{.}\;\|v{\|}_{H^{1}\left(\Omega \setminus \Sigma ;R^{d}\right)}^{2}\leq C_{K}\int_{\;\;\Omega \setminus \Sigma }\left(|v{|}^{2}+|e\left[v\right]{|}^{2}\right)\;dx\;\;\;\;\text{for}\;v\in V\left(0;\Sigma \right).$$

**Definition B.1** (Crack evolution). *If* ${\left\{\Sigma \left(t\right)\right\}}_{t_{0}\leq t\leq t_{1}}$ *satisfies the following conditions, we call it a crack evolution in* Ω . 1) $\Sigma \left(t\right)\in C_{0}\;\left(t_{0}\leq t<t_{1}\right)$ , $\Sigma \left(t_{1}\right)\in C$ . 2) $\Sigma \left(t\right)\subset \Sigma \left(\overset{˜}{t}\right)\;\left(t_{0}\leq t\leq \overset{˜}{t}\leq t_{1}\right)$ . 3) $H^{d-1}\left(\Sigma \left(t\right)\right)=H^{d-1}\left(\Sigma \left(\overset{˜}{t}\right)\right)$ *implies* $\Sigma \left(t\right)=\Sigma \left(\overset{˜}{t}\right)$ . *Furthermore*, *if* $H^{d-1}\left(\Sigma \left(t\right)\right)$ *is continuous in* $t\in [t_{0},t_{1}]$ , *then* ${\left\{\Sigma \left(t\right)\right\}}_{t_{0}\leq t\leq t_{1}}$ *is called a continuous crack evolution in* Ω .

**Definition B.2** (Crack path). *If* ${\left\{\Sigma_{p}\left(l\right)\right\}}_{l_{0}\leq l\leq l_{1}}$ *satisfies the following conditions, we call it a crack path in* Ω . 1) $\Sigma_{p}\left(l\right)\in C_{0}\left(l_{0}\leq l<l_{1}\right)$ , $\Sigma_{p}\left(l_{1}\right)\in C$ . 2) $\Sigma_{p}\left(l\right)\subset \Sigma_{p}\left(\overset{˜}{l}\right)$ $\left(l_{0}\leq l\leq \overset{˜}{l}\leq l_{1}\right)$ . 3) $l=H^{d-1}\left(\Sigma_{p}\left(l\right)\right)\;\left(l_{0}\leq l\leq l_{1}\right)$ .

**Proposition B.3** ([13]). *If* ${\left\{\Sigma \left(t\right)\right\}}_{t_{0}\leq t\leq t_{1}}$ *is a continuous crack evolution in* Ω , *then there exists a unique crack path* ${\left\{\Sigma_{p}\left(l\right)\right\}}_{l_{0}\leq l\leq l_{1}}$ *in* Ω *such that* $\Sigma \left(t\right)=\Sigma_{p}\left(L\left(t\right)\right)$ *for* $t\in [t_{0},t_{1}],$ *where* $L\left(t\right):=H^{d-1}\left(\Sigma \left(t\right)\right)$ .
